# Incorporation of Nonyl 3,4-Dihydroxybenzoate Into Nanostructured Lipid Systems: Effective Alternative for Maintaining Anti-Dermatophytic and Antibiofilm Activities and Reducing Toxicity at High Concentrations

**DOI:** 10.3389/fmicb.2020.01154

**Published:** 2020-06-05

**Authors:** Caroline Barcelos Costa-Orlandi, Aline Serafim-Pinto, Patrícia Bento da Silva, Níura Madalena Bila, Jean Lucas de Carvalho Bonatti, Liliana Scorzoni, Junya de Lacorte Singulani, Claudia Tavares dos Santos, Ana Carolina Nazaré, Marlus Chorilli, Luis Octávio Regasini, Ana Marisa Fusco-Almeida, Maria José Soares Mendes-Giannini

**Affiliations:** ^1^School of Pharmaceutical Sciences, Department of Clinical Analysis, Universidade Estadual Paulista (UNESP), Araraquara, Brazil; ^2^School of Pharmaceutical Sciences, Department of Drugs and Medicines, Universidade Estadual Paulista (UNESP), Araraquara, Brazil; ^3^Universidade Eduardo Mondlane, School of Veterinary, Maputo, Mozambique; ^4^Institute of Biosciences, Humanities and Exact Sciences, Department of Chemistry and Environmental Sciences, Universidade Estadual Paulista (UNESP), São José do Rio Preto, Brazil

**Keywords:** nanoparticles, dermatophytes, *Trichophyton rubrum*, *Trichophyton mentagrophytes*, alternative models, *Caenorhabditis elegans*, zebrafish, biofilms

## Abstract

Dermatophytosis is the most common mycosis worldwide, affecting approximately 20 to 25% of the population, regardless of gender, race, color, and age. Most antifungal agents used for the treatment of dermatophytosis belong to the azole and allylamine classes. Dermatophytes are reported to be resistant to most commercial drugs, especially microbial biofilms, in addition to their considerable toxicity. It should be emphasized the importance of looking for new molecules with reduced toxicity, as well as new targets and mechanisms of action. This work aims to incorporate nonyl 3,4-dihydroxybenzoate, a potent fungicide compound against planktonic cells and dermatophyte biofilms in nanostructured lipid systems (NLS), in order to reduce toxicity in high concentrations, improve its solubility and maintain its effectiveness. The compound was incorporated into NLS constituted by cholesterol, mixture of polyoxyethylene (23) lauryl ether (Brij^®^98) and soybean phosphatidylcholine (Epikuron^®^ 200)], 2: 1 ratio and PBS (phosphate-buffered saline). The characterization of the incorporation was performed. Susceptibility tests were conducted according to document M38-A2 by CLSI (2008). The toxicity of the NLS compound was evaluated in HaCaT cell lines by the sulforhodamine B method and in alternative models *Caenorhabditis elegans* and zebrafish. Finally, its efficacy was evaluated against the mature *Trichophyton rubrum* and *Trichophyton mentagrophytes* biofilms. NLS and nonyl 3,4-dihydroxybenzoate loaded into NLS displayed sizes ranging from 137.8 ± 1.815 to 167.9 ± 4.070 nm; the polydispersity index (PDI) varying from 0.331 ± 0.020 to 0.377 ± 0.004 and zeta potential ranging from −1.46 ± 0.157 to −4.63 ± 0.398 mV, respectively. Polarized light microscopy results confirmed the formation of NLS of the microemulsion type. Nonyl incorporated into NLS showed minimum inhibitory concentration (MIC) values, ranging from 2 to 15.6 mg/L. The toxicity tests presented cell viability higher than 80% in all tested concentrations, as well as, a significantly increased of the survival of *Caenorhabditis elegans* and zebrafish models. Anti-biofilm tests proved the efficacy of the incorporation. These findings contribute significantly to the search for new antifungals and allow the systemic administration of the compound, since the incorporation can increase the solubility of non-polar compounds, improve bioavailability, effectiveness and reduce toxicity.

## Introduction

About 1.2 billion people worldwide are estimated to suffer from some fungal diseases (Denning and Bromley, 2015) and most of these diseases cause infections of the skin or mucosa, usually chronic. Among the agents of these infections are dermatophytes that have a predilection for keratinized tissues (skin, hair and nails) of human and animals, producing a dermatophytosis, also popularly known as “ringworm” or tinea (Weitzman and Summerbell, 1995; Costa-Orlandi et al., 2012; Bouchara et al., 2017). Despite the proposal for a new phylogenetic taxonomy that classifies dermatophytes into six clades (*Trichophyton*, *Epidermophyton*, *Nannizzia*, *Microsporum*, *Lophophyton*, and *Arthroderma*), the anamorphic genera *Trichophyton*, *Microsporum*, and *Epidermophyton* remain the most important (de Hoog et al., 2017; Zhan and Liu, 2017). Dermatophytosis is considered the most common mycosis worldwide, affecting approximately 20 to 25% of the population, regardless of sex, race, color, and age (Havlickova et al., 2008; Zhan and Liu, 2017).

For the treatment of dermatophytosis there is a reasonable amount of available drugs, however, the vast majority belong to the families of the azoles and alylamines (Gupta and Cooper, 2008; Martinez-Rossi et al., 2008; Gupta et al., 2017). For lesions in early stages, topical treatment is the most suitable and the main drugs administered are cyclopyrox, amorolfine, clotrimazole, miconazole, econazole, tioconazole, naftifine and terbinafine (Kaur et al., 2008; Gupta et al., 2017). Although terbinafine is the gold standard for the treatment of dermatophytosis, new topical drugs have been developed and recently approved by the FDA (Food and Drug Administration) (Gupta and Simpson, 2012). Among them, luliconazole (Feng et al., 2014), efinaconazole (Tosti, 2013), and tavaborole (Zane et al., 2015), proved to be safe and effective (Zeichner, 2015). Topical therapy is usually non-toxic, since systemic absorption tends to be minimal, which eliminates the possibility of drug interactions (Gupta et al., 2017).

In the treatment of onychomycosis, topical antifungals have low efficacy when administered alone or in combination with other systemic antifungals. The association of topical and systemic drugs still used, such as terbinafine, can result in a faster cure and is indicated in the case of extensive or chronic lesions and in onychomycosis and tinea capitis (Kaur et al., 2008). The systemic drugs most used nowadays are still fluconazole, ketoconazole, itraconazole, terbinafine, and griseofulvin (Gupta and Cooper, 2008; Hay, 2009; Gupta et al., 2017). In onychomycosis, griseofulvin and ketoconazole are no longer used due to the development of resistance and hepatotoxicity, respectively (Gupta et al., 2013). There has been no progress in the development of new antifungals with anti-dermatophyte activity with oral administration route in recent years (Gupta et al., 2017). Although newer oral drugs, such as pramiconazole, ravuconazole, and albaconazole have been tested *in vitro* or by phase 1 to 2 clinical trials in dermatophyte infections (tinea corporis and onychomycosis), at present, they are not marketed for these indications (Hay, 2018). Even with this available arsenal, recurrences and reinfections are of great concern, especially in tinea pedis and onychomycosis, with reports of variation between 10 and 50% (Gupta et al., 2016, 2017).

Biofilms are highly organized communities of microorganisms formed by one or multi-species (from the functional and structural point of view) adhered to each other or to a surface and surrounded by a polymeric extracellular matrix that they themselves produce (Ramage et al., 2009; Costa-Orlandi et al., 2014; Costa-Orlandi et al., 2017). These communities protect microorganisms from action against the environment and against antimicrobials, increase microbial communication and virulence, in addition to promoting metabolic cooperation and the emergence of a community based on the regulation of gene expression (Percival et al., 2012; Costa-Orlandi et al., 2017).

In 2014, our group demonstrated for the first time the ability of dermatophytes to form biofilms (Costa-Orlandi et al., 2014). This phenomenon is of great importance and concern as it is associated with resistance to antifungal therapy, as well as the high rates of relapse of dermatophytosis. Once confirmed the resistance and the need of using high concentrations (and possibly toxic) of drugs for these communities to be eradicated, it is imperative to search for new compounds with antifungal properties and, mainly, antibiofilm.

Therefore, the compound nonyl 3,4-dihydroxybenzoate is a derivative of protocatechuic acid (acid 3,4-dihydroxybenzoic) with nine carbons in the side alkyl chain and has shown excellent activity against several fungi, among them dermatophytes (Soares et al., 2014), *Paracoccidioides brasiliensis*, *Paracoccidioides lutzii* (Medina-Alarcón et al., 2017), *Cryptococcus neoformans*, *Cryptococcus gattii*, and *Histoplasma capsulatum* (unpublished data). In addition to antifungal activity, protocatechuic acid, together with its natural and synthetic derivatives, has been associated with a wide range of biological actions (Soares et al., 2014). Against dermatophytes, this compound showed excellent activity, in addition to fungicidal behavior, both in planktonic cells and in biofilms formed by these fungi, even the latter requiring higher concentrations for eradication, which have a certain toxicity in normal human keratinocytes.

Faced with a fungicidal compound, with excellent activity against planktonic cells and biofilms and with a broad spectrum of action, it is essential to seek alternatives for the reduction of toxicity in the active concentrations against biofilms.

In recent years, nanotechnology is a tool used widely by researchers in order to reduce toxicity, improve efficacy, specificity, tolerability, and therapeutic index of bioactive compounds. Several strategies can be used in nanotechnology such as liposomes, polymeric nanoparticles, nanostructured lipid systems (NLS), magnetic nanoparticles, among others. Among the NLS, we highlight the microemulsions (MEs), which can be defined as transparent emulsions, in which an oil is dispersed in an aqueous medium (or vice versa), containing a surfactant, associated or not with an appropriate co-surfactant, generating a thermodynamically stable system and presenting droplets of the internal phase in the order of nanometers (de Freitas et al., 2014). The bioactive compound can be served in the microemulsions when solubilized in the oil or aqueous phase.

Therefore, this work aims to incorporate nonyl 3,4-dihydroxybenzoate, a potent fungicidal compound against planktonic cells and dermatophyte biofilms in nanostructured lipid systems, of the type microemulsion, in order to reduce toxicity at high concentrations as well as improve their solubility and maintain their efficacy.

## Materials and Methods

### Microorganisms

In this work, strains of *Trichophyton rubrum* ATCC 28189, *T*. *rubrum* ATCC MYA-4438 and *Trichophyton mentagrophytes* ATCC 11481 were used. All strains belong to the collection of the Clinical Mycology Laboratory of the Department of Clinical Analysis, of the School of Pharmaceutical Sciences at UNESP. The dermatophytes were maintained on Potato Agar Dextrose (Difco, BD Biosciences) and incubated at 28°C for 7 days or until there was sporulation (Costa-Orlandi et al., 2014).

### Synthesis and Incorporation of Nonyl 3,4-Dihydroxybenzoate to the Nanostructured Lipid System (NLS)

The nonyl compound was synthesized according to Soares et al. (2014). Incorporation into the nanostructured lipid system was performed according to Bonifácio et al. (2015), with minor modifications. The formulations were prepared by adding the oil phase (10% cholesterol) to the surfactant [10% mixture of polyoxyethylene (23) lauryl ether (Brij^®^98) and soybean phosphatidylcholine (Epikuron^®^ 200)], 2: 1 ratio, followed by the aqueous phase (phosphate-buffered saline −80% PBS). The mixture was sonicated (Q500, QSonica^®^) with a power of 500 watts and 20% amplitude, for 8–10 min in an ice bath and discontinuously, with an interval of 30 s every minute. In order to remove the titanium residues released by the sonicator rod, the formulations were centrifuged at 11,180 (x g) for 15 min, obtaining the NLS’s. Then, in transparent flasks of penicillin, 60 mg of the compound was added in 2 mL of NLS and homogenized for 2 min in an amplitude of 15%, discontinuously, in an ice bath, so that the material could be incorporated into the nanosystem at a concentration of 30,000 mg/L.

### Physical–Chemical Characterization of Nanostructured Systems

#### Determination of the Average Diameter of Nanoparticles Using the Dynamic Light Scattering Method and the Zeta Potential (ZP)

The determination of the particle diameter was carried out according to the photon correlation methodology, described by Silva et al. (2009), through the laser radiation dynamic spreading equipment – Light Scattering – Brookhaven. Droplets of the samples were placed in an analysis chamber so that a laser beam (λ = 530 nm) would cross the entire droplet dispersion, at a temperature of 20°C. When a beam of light passes through a colloidal dispersion, the particles or drops spread the light in all directions, being possible to observe time-dependent fluctuations in the intensity of the scattering through a detector that processes the data and sends it to a computer, determining the polydispersity index (PDI). From this technique, the hydrodynamic radius of the colloidal particles of the Einstein-Stokes equation is calculated, in which D = particle diffusion coefficient; K = Boltzmann constant (1.3807 × 10^–23^ Nm K^–1^); T = absolute temperature (293, 15 K); π = 3.141592; η = viscosity (1.002 × 10^–3^ NM-2s); Rh = hydrodynamic radius. Ten determinations of the diameter and polydispersity index (PDI) of the droplets of each sample were performed.

$$D=\frac{K\,T}{6\,\pi \,\eta \,R\,h}$$

The zeta potential (ZP) was analyzed in order to determine the surface charge of the lipid systems incorporated or not with the nonyl, from the electrophoretic mobility, using Zetasizer Nano NS equipment (Malvern Instruments, Malvern, United Kingdom). The samples were previously diluted (10 μL/mL) in aqueous potassium chloride solution to maintain an ionic strength constant during the analysis and subsequently placed in the electrophoretic cell in order to determine the surface potential, by calculating the mean and standard deviation (*n* = 3).

#### Polarized Light Microscopy

In order to investigate the homogeneity of the dispersions, areas of anisotropy were analyzed with the aid of polarization. A small amount of the formulations were placed on glass slides, covered with coverslips and analyzed under a polarized light microscope Lx 400P – Labomed^®^.

### *In vitro* Susceptibility Test of Dermatophytes to Nanostructured Compound and Determination of Minimum Fungicide Concentration

The susceptibility tests were conducted as described in document M38-A2, proposed by the Clinical and Laboratory Standards Institute CLSI (2008), with minor modifications.

#### Dilution of Incorporated and Non-incorporated Compound and Antifungal Drugs

Nonyl incorporated into the NLS was diluted aseptically in RPMI-1640 medium with L-glutamine, without sodium bicarbonate, using phenol red as a pH indicator (Gibco^®^) and buffered with MOPS – [3- (*N*-morpholine) propanesulfonic acid] (Sigma-Aldrich), pH = 7. The unincorporated compound was diluted in DMSO (stock solution) in a concentration of 30,000 mg/L, so that the final concentration of DMSO in the initial working solution (in RPMI medium) was less than 1%, not compromising the test. Calculations were performed in order to obtain an initial concentration of 250 mg/L in the microdilution plate (96 wells) and a final concentration of 0.06 mg/L. As controls, NLS without incorporation and nonyl protocatechuate were tested at the same concentrations, as well as antifungal drugs. With respect to drugs, stock solutions of terbinafine and fluconazole were prepared considering their purity, through calculations recommended in document M38-A2. The concentration ranges for fluconazole tested were 64 to 0.125 mg/L and for terbinafine, 0.5 to 0.001 mg/L. One hundred microliters of each dilution were distributed in the microdilution plate wells.

#### Inoculum Preparation

The inoculum was prepared by subculturing dermatophytes on potato dextrose agar (Difco, BD biosciences), followed by incubation at 28–30°C for 7 days. Five milliliters of sterile 0.85% saline were added to the cultures and, with the aid of a sterile swab, the conidia were detached from the hyphae, forming a suspension. The inocula were prepared in RPMI-1640 medium and adjusted after counting the conidia on the hemocytometer, in order to obtain a final concentration in the microdilution plate corresponding to 2.5 × 10^3^ cells/mL. Then, 100 μL of the inoculums were distributed in the microdilution plate wells, obeying the media sterility controls (negative, with 200 μL of RPMI-1640 medium) and the growth control (positive, containing 100 μL of RPMI and 100 μL of the inoculum). Finally, the plates were incubated, with shaking, at 35°C for 120 h. Visual readings were performed, and all tests were performed with three independent experiments. The *T. rubrum* strain ATCC MYA-4438 was used as a test quality control.

#### Minimum Fungicide Concentration

To determine the minimum fungicidal concentration (MFC), an aliquot of the content of each well of the microdilution plates was subcultured into plates containing potato dextrose agar. The plates were incubated at 35°C for up to 120 h, without shaking. The MFC is defined as the lowest concentration of the compound, nanoparticle or drug where the development of microorganisms does not occur (Soares et al., 2014; Ghannoum et al., 2015).

### Evaluation of Toxicity of Nonyl Nanostructures in Epithelium Cells by the Sulforodamine B Method

After certifying the antifungal activity, as well as the incorporation stability, toxicity tests on human skin keratinocytes (HaCat ATCC^®^ PCS-200-011^TM^) were performed in order to verify the possibility of reducing the toxicity of the highest concentrations of nonyl incorporated. After obtaining 90% confluence, the cells were trypsinized, resuspended in DMEM medium (Gibco) and counted on the hemocytometer, in order to obtain a concentration of 1 × 10^5^ cells per well in the microdilution plate. One hundred microliters of the HaCat suspensions were dispensed in 96-well plates and incubated at 37°C with 5% CO_2_ tension. After 24 h of incubation, the supernatant was removed and 100 μL of the several concentrations of nonyl incorporated into the NLS were prepared in DMEM medium and placed in contact with the cells. The plates were re-incubated for 24 h. Cell growth was assessed using the colorimetric method of sulforhodamine B (Sigma-Aldrich), according to the manufacturer’s recommendations (Escobar et al., 2009).

### Toxicity Assays on *C. elegans* Model

For the toxicity assay in *Caenorhabditis elegans*, the wild type strain (N2) and the mutant strain AU37 [*glp-4*(*bn2*) I; *sek-1*(*km4*) X] were used after synchronization in stage L4, of young adults. About 20–30 larvae were transferred to each well of 96-well plates containing 100 μL of a medium composed of 60% 50 mM NaCl; 40% BHI broth; 10 mg/mL of cholesterol in ethanol; 200 mg/mL ampicillin and 90 mg/mL kanamycin. Then, 100 μL of dilutions of nonyl incorporated or not to the NLS with final concentrations in each well ranging from 1.98 to 500 mg/L were added. The plates were incubated at 25°C for 24 h. Survival was assessed by nematode mobility and shape (stick-shaped larvae were considered dead, while sinusoidal larvae were considered alive) in optical microscope on 40X objective lens (Scorzoni et al., 2013; Ahn et al., 2014; Gupta et al., 2015; Gonzalez-Moragas et al., 2017; Medina-Alarcón et al., 2017; Abdullah et al., 2019). Images were acquired using the equipment In cell Analyzer (GE healthcare) on 10X objective lens.

### Teratogenicity Assay in Zebrafish Embryos (*Danio rerio*)

The zebrafish (wild type) were maintained according to the standard protocol (28 ± 0.5°C with 14:10 day/night photoperiodism). For embryo collection, adult fish were placed for mating in an appropriate tank, in a male/female ratio of 1: 1, 1: 2 or 2: 1, overnight. The next day, the embryos were collected, washed with embryonic medium (10 mM NaCl, 0.34 mM KCl, 0.66 mM CaCl_2_.2H_2_O, 0.66 mM MgCl_2_.6H_2_O) and selected for the test. The selected embryos were transferred to the 96-well plates (two embryos per well). Nonyl incorporated and not incorporated into NLS, as well as the NLS without incorporation were diluted in embryonic medium and prepared in order to be tested in the concentration range from 1.95 to 250 mg/L. Embryos not submitted to treatment were used as controls. The plates were incubated at 28°C and malformation phenotypes were observed after 6, 24, 48, and 72 h post-fertilization (hpf). The effect of compound concentrations on embryo development was assessed with the aid of a magnifying glass equipment *Carl Zeiss Stereo Discovery* (Kimmel et al., 1995; Busquet et al., 2014; Li et al., 2016; Palanco et al., 2017).

### Effect of Nanostructured Nonyl Against Preformed Biofilms

Biofilms were formed in 96-well plates and incubated until maturation, as described by Costa-Orlandi et al. (2014). Briefly, a fungal suspension was prepared in sterile 0.85% saline at a concentration of 1 × 10^6^ cells/mL. Approximately 200 μL were dispensed on the plates, which were then incubated for 4 h at 35–37°C without shaking, in order to occur the biofilms pre-adhesion. The supernatant was removed, and the wells washed with sterile PBS to remove non-adherent cells. The RPMI medium was added and the plates were re-incubated at 37°C for 72 h (maturation period), with agitation of 150 rpm. After 72 h, the biofilm supernatants were again removed and the working solutions (500–0.98 mg/L) of the nanostructured nonyl were placed in contact with the biofilms, together with their respective controls. The plates were incubated for another 72 h. Metabolic activities were assessed by the XTT reduction assay, as previously standardized (Martinez and Casadevall, 2006; Pierce et al., 2008; Costa-Orlandi et al., 2014). The reduction in metabolism by at least 50% was compared to the biofilm growth control, which corresponds to 100%, free of treatment.

### Scanning Electron Microscopy (SEM)

The mature biofilms of the *T. rubrum* strain ATCC 28189 were formed according to item 2.8, but in 24-well plates. One thousand microliters of the fungal suspension were placed in each well. Once the pre-adhesion and maturation periods were established, the mature biofilms were treated with the working solution of nanostructured nonyl in the highest concentration previously tested (500 mg/L). The plates were incubated for another 72 h with shaking at 150 rpm. After the established times, the supernatants were removed, the biofilms were washed with PBS and fixed with 800 μL of 2.5% glutaraldehyde solution (Sigma-Aldrich) for 1 to 2 h at 4°C. The glutaraldehyde solution was removed from the wells, then the biofilms were dehydrated with increasing concentrations of alcohol (50–100%). After drying, the bottom of the plates was cut with the aid of a scalpel and the samples were mounted on aluminum cylinders containing carbon strips and evaporated under high vacuum (Denton Vacuum Desk V, Jeol United Kingdom) for the gold coating. Topographical characteristics of biofilms were analyzed by electron microscope Jeol 6610LV scanning JSM- at the School of Dentistry of UNESP at Araraquara, SP, Brazil (Martinez et al., 2010; Costa-Orlandi et al., 2014).

### Colony Forming Units per Milliliter Assay (CFU/mL)

For the CFU counting test, biofilms were formed in the 96-well plates and treated as described in item 2.8 with the highest concentrations of the incorporated compound (250 and 500 mg/L). The contents of the wells were transferred to 1.5 mL microtubes (Kasvi) and vortexed until complete disintegration of the biofilm cells. Serial dilutions were performed in PBS and 30 μL aliquots of the diluted content were plated in 90 × 15 mm Petri dishes, containing sterile glass beads and potato agar dextrose (Difco, BD biosciences). The plates were incubated at 28°C, until the growth of colony forming units (Santos et al., 2016).

### Statistical Analysis

Statistical analysis was performed using analysis of variance (one-way ANOVA) with Bonferroni post-test using the GraphPad Prism 5 software. Values of *p* < 0.05 were considered statistically significant. All tests were performed at least in triplicate and in three independent experiments.

## Results

### Characterization of the Physical–Chemical Properties of the Nanostructured Lipid System

The average diameter of the NLS particles was 137.8 ± 1.815 nm. The incorporation of nonyl in the NLS caused a small variation in the size of the particles, 167.9 ± 4.070 nm. The polydispersity index (PDI) shows the relative homogeneity between the particle sizes distributed in the measured sample. The PDI values varied between 0.331 ± 0.020 and 0.377 ± 0.004, presenting an average range of variation between the control NLS and the NLS containing the incorporated compound. Regarding the zeta potential (ZP), the result for the control NLS was −1.46 ± 0.157 mV, while for the formulation containing the incorporated compound, −4.63 ± 0.398 mV.

**FIGURE 1:**
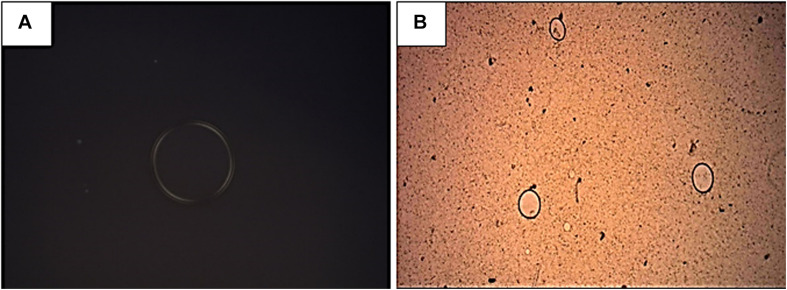
Photomicrograph of the nanostructured lipid system (NLS) in dark field **(A)** and of the system incorporated with nonyl protocatechuate (NLS + nonyl) in bright field **(B)**, in 4X magnification. An isotropy of the samples can be observed, suggesting formation of the micro-emulsified system.

Figures 1A,B show the photomicrographs of the polarized light microscopy in dark field (Figure 1A) and bright field (Figure 1B) of the formulations without (NLS control) and with the incorporation (NLS + nonyl), respectively. The samples show an isotropic behavior, i.e., in the plane of polarized light, there is no light deviation, suggesting the formation of micro-emulsified system.

### Susceptibility Test of Dermatophytes Against Nanostructured Compound and Minimum Fungicide Concentration (MFC)

The minimum inhibitory concentration (MIC) values of the formulations and their respective controls are shown in Table 1. The NLS incorporated with nonyl inhibited the growth of the three strains tested at concentrations ranging from 2 to 15.6 mg/L. These values were 2 to 8 times higher when compared to the MIC values of unincorporated nonyl (1–2 mg/L). The nanosystem without the incorporation showed no activity on the tested fungi. With respect to drugs, fluconazole showed activity against *T. mentagrophytes* ATCC 11481 (MIC = 1 mg/L) and resistance against *T. rubrum* ATCC 28189 (MIC = 64 mg/L). Terbinafine showed the best results against all strains tested in planktonic form, with MIC values in the ranges from 0.03 to 0.0075 mg/L. The MIC values of both drugs for *T. rubrum* strain ATCC MYA-4438 corresponded to the test quality control values recommended in document M38-A2 (MIC = 4 mg/L for fluconazole and MIC = 0.03 mg/L for terbinafine), ensuring the quality of the test (CLSI, 2008).

**TABLE 1:** Minimum inhibitory concentration (MIC) and minimum fungicidal concentration (MFC) values expressed in mg/L for nonyl 3,4-dihydroxybenzoate incorporated or not into the nanostructured lipid system (NLS) and antifungal drugs.

**Fungi/compounds and NLS**	***T. rubrum* ATCC 28189**		***T. mentagrophytes* ATCC 11481**		***T. rubrum* ATCC MYA-4438**	
	
	**MIC**	**MFC**	**MIC**	**MFC**	**MIC**	**MFC**
NLS	>250	>250	>250	>250	>250	>250
NLS + nonyl	2	2	15.6	15.6	4	4
Nonyl	1	1	2	2	2	2
Fluconazole	64	>64	1	4	4	–
Terbinafine	0.03	0.03	0.0075	0.0075	0.03	–

Tests of MFC showed that the incorporation of the compound in the NLS maintained the fungicidal activity, previously investigated (Soares et al., 2014) with MFC equal to MIC values (Table 1).

### Evaluation of Cytotoxicity of Formulations in Human Epithelium Monolayers

**FIGURE 2:**
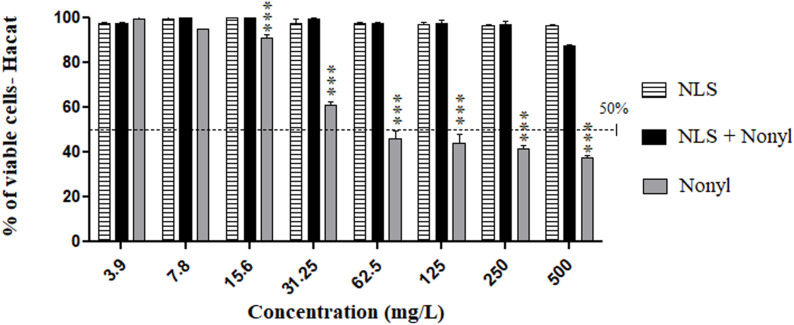
Cytotoxicity of NLS and nonyl incorporated or not into NLS in HaCat cell line. There was a significant reduction in toxicity when non-incorporated nonyl was compared to the same compound incorporated into the nanostructured lipid system. ***p < 0.0001.

The epithelial cells treated with the nanostructured nonyl, as well as with the control formulation, showed viability greater than 80% at all concentrations (3.9–500 mg/L) tested in the cytotoxicity tests (Figure 2). These results are promising, since treatment with nonyl in the same epithelial cells resulted in a viability of only 30% at a concentration of 62.5 mg/L. Thus, it is observed that the incorporation of the compound in nanostructure decreased the cytotoxicity becoming non-toxic to human epithelial cells.

### *In vivo* Toxicity Assays

#### *C. elegans* Model

**FIGURE 3:**
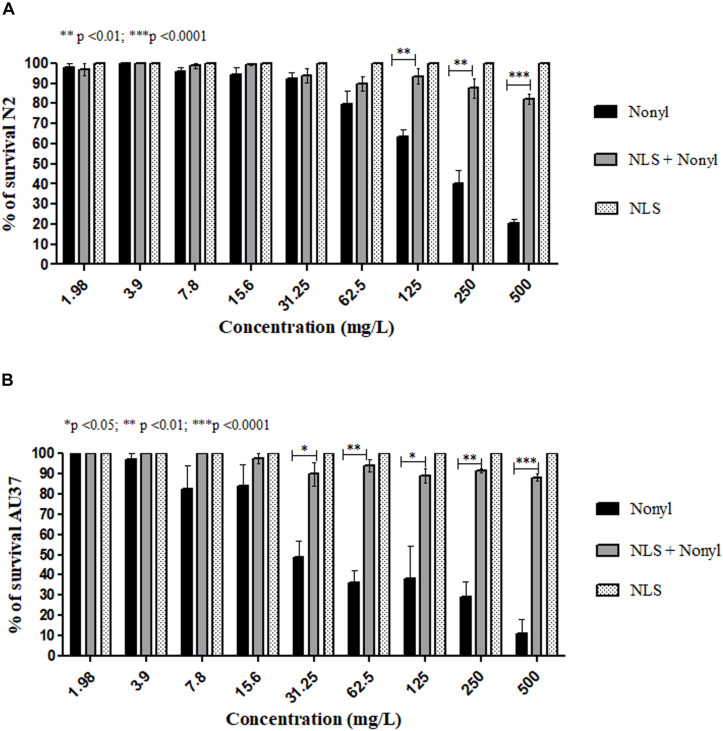
Survival percentage of *Caenorhabditis elegans* wild type N2 **(A)** and mutant AU37 strains **(B)** after 24 h treatment with different concentrations of nonyl incorporated or not into the nanostructured lipid system. Larvae survival increased when nonyl was incorporated into the NLS in both strains.

**FIGURE 4:**
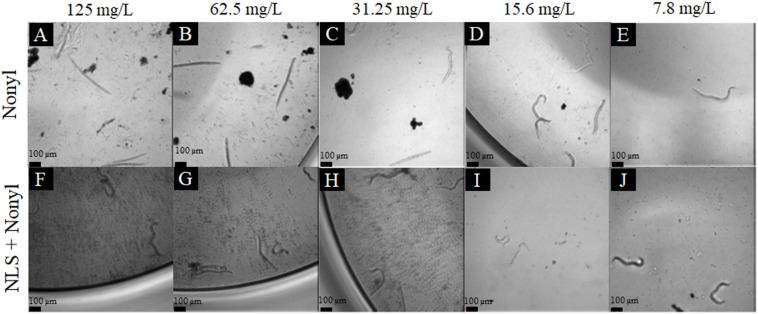
Representative image of *C. elegans* AU37 larvae treated with nonyl 125 mg/L **(A)**; 62.5 mg/L **(B)**; 31.25 mg/L **(C)**; 15.6 mg/L **(D)**; 7.8 mg/L **(E);** and NLS + nonyl 125 mg/L **(F)**; 62.5 mg/L **(G)**; 31.25 mg/L **(H)**; 15.6 mg/L **(I)**; 7.8 **(J)** (10X magnification). At the highest concentrations, the larvae treated with the nanostructured nonyl remained alive, while those treated with non-incorporated nonyl died (stick-shaped larvae were considered dead, while sinusoidal larvae were considered alive).

The results of the toxicity tests on *C. elegans* are shown in Figure 3. Figure 3A shows the percentage of survival of the larvae of the wild type strain N2 after contact with different concentrations of the compound incorporated or not. Concentrations equal to or greater than 250 mg/L of the free nonyl were able to kill more than 50% of the larvae. Regarding to nonyl incorporated into the NLS, toxicity was significantly reduced when compared to treatment with the compound not incorporated in the concentrations of 125 mg/L (*p* < 0.01), 250 mg/L (*p* < 0.01) and in concentration of 500 mg/L (*p* < 0.0001). In addition, in all tested concentrations also exhibited, a survival greater than 80%, demonstrating the decrease in toxicity. For the AU37 mutant strain (Figure 3B), concentrations from 31.25 mg/L of non-incorporated nonyl showed significant toxicity. In contrast, the incorporated nonyl showed a survival percentage greater than 80%, in all tested concentrations. When compared to the non-incorporated compound, treatment with nonyl + NLS significantly increased larval survival at concentrations of 31.25 mg/L (*p* < 0.05); 62.5 mg/L (*p* < 0.01); 125 mg/L (*p* < 0.05); 250 mg/L (*p* < 0.01) and, at a concentration of 500 mg/L (*p* < 0.0001). It is also observed that for both strains, the NLS controls did not show any toxicity to the larvae. It is concluded, therefore, that the incorporation significantly reduced the toxicity of the compound, increasing the survival of the larvae of both strains. Figure 4 represents the larvae of strain AU37 treated with higher and lower concentrations of nonyl alone (A–D) and incorporated (E–I). At the highest concentrations, the larvae treated with the nanostructured nonyl remained alive, while those treated with non-incorporated nonyl died.

#### Zebrafish (*D. rerio*) Model

**FIGURE 5:**
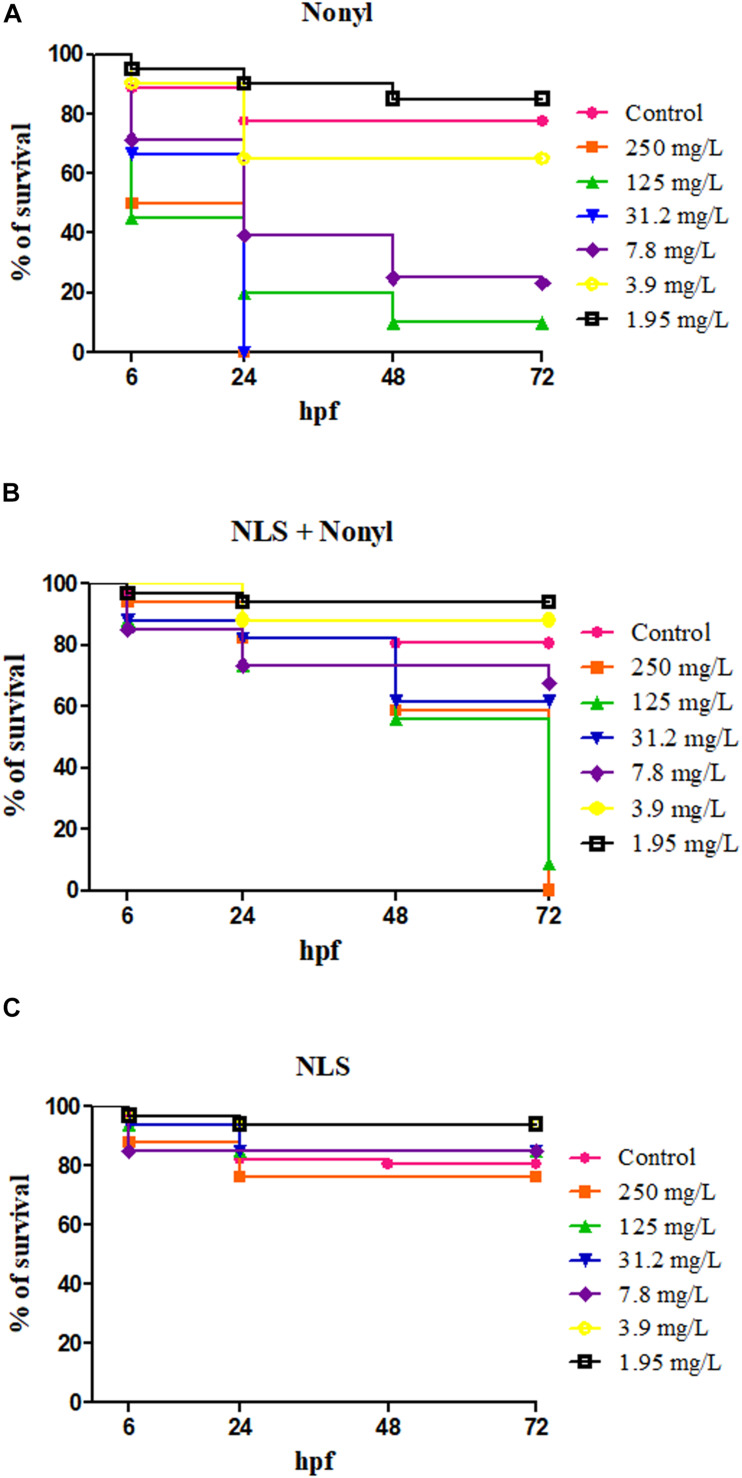
Survival curve of zebrafish embryos in contact with different concentrations, expressed in milligrams per liter (mg/L) of nonyl **(A)**, nonyl incorporated into NLS **(B)** and NLS without the incorporation **(C)** after 6, 24, 48, and 72 h post-fertilization (hpf). The zebrafish embryos placed in contact with the nonyl incorporated to NLS showed a higher survival rate compared to the embryos placed in contact with the nonyl alone. The nanostructured system alone maintained embryos alive.

The lowest concentrations of nonyl tested (1.95 and 3.9 mg/L) did not produce considerable toxic effects on zebrafish embryos until 72 h after fertilization (hpf) (Figure 5A). Clearly, the toxic effect of the compound on embryonic development increased proportionally to the concentration. The LC_50_ (lethal concentration 50%) of non-incorporated nonyl was calculated, corresponding to 6.2 mg/L, very close to the MIC region (1–2 mg/L), which indicates that even at low MIC concentrations, the compound is highly teratogenic for zebrafish embryos.

After incorporation of nonyl, there was a significant reduction in the phenotypes of malformation of embryos at concentrations below 125 mg/L (Figure 5B) and even at the highest concentration (250 mg/L). The incorporation led to a later toxicity in the embryos (72 h) when compared to non-incorporated nonyl (24 h) and the lethal concentration 50 increased to 79.2 mg/L, about 12.7 times.

The formulation without the incorporation was tested as a control and the results showed a high percentage of survival in all corresponding concentrations (Figure 5C).

### Effect of Nanostructured Nonyl Against Mature Biofilms

**FIGURE 6:**
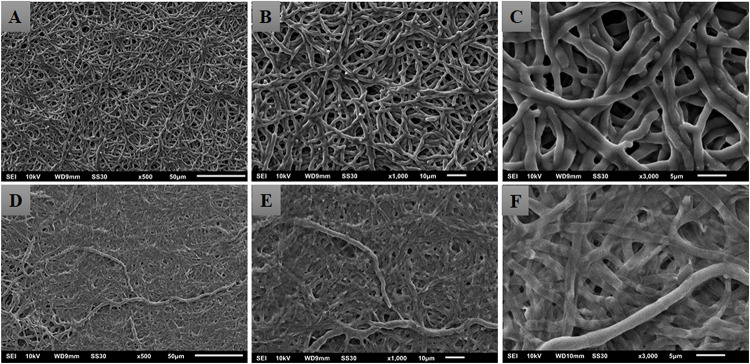
Electromicrographs of *Trichophyton rubrum* ATCC 28189 mature biofilms untreated **(A–C)** and treated with nonyl incorporated into the lipid nanosystem at a dose of 500 mg/L **(D–F)**. The incorporated nonyl caused an extravasation of cytoplasmic content resulting in collapse of the walls of the hyphae.

When using the XTT reduction assay to quantify the metabolic activities of biofilms treated with nonyl incorporated into the NLS, the results in Table 2 showed that, unlike the non-incorporated nonyl (SMIC_50_ = 125–250 mg/L), surprisingly, the incorporated one was not able to reduce the metabolic activities of mature biofilms at the maximum tested concentrations (SMIC_50_ > 500 mg/L). In order to confirm the lack of activity or any limitation of the XTT reduction assay, scanning electron microscopy (SEM) was performed to check possible damage to the biofilm topographies (Figure 6).

**TABLE 2:** Sessile minimum inhibitory concentration values, expressed in mg/L, of nonyl and nonyl incorporated into nanostructured lipid system (NLS) capable of reducing at least 50% of the metabolic activities of mature *T. rubrum* and *T. mentagrophytes* biofilms.

***T. rubrum* ATCC MYA 4438**	***T. rubrum* ATCC MYA 4438**	***T. rubrum* ATCC 28189**	***T. rubrum* ATCC 28189**	***T. mentagrophytes* ATCC 11481**	***T. mentagrophytes* ATCC 11481**
Nonyl	Nonyl + NLS	Nonyl	Nonyl + NLS	Nonyl	Nonyl + NLS
SMIC_50_	SMIC_50_	SMIC_50_	SMIC_50_	SMIC_50_	SMIC_50_
250	>500	125	>500	125	>500

*SMIC_50_ (Sessile minimum inhibitory concentration capable of reducing at least 50% of the metabolic activities of mature biofilms).*

The electromicrographs obtained from SEM revealed a totally different behavior from that observed in the XTT assay. The treatment with the incorporated nonyl caused a total collapse of the hyphae walls, resulting in a probable leakage of the cytoplasmic content. Rare intact hyphae were seen interlaced with the damaged hyphae (Figures 6D–F).

**FIGURE 7:**
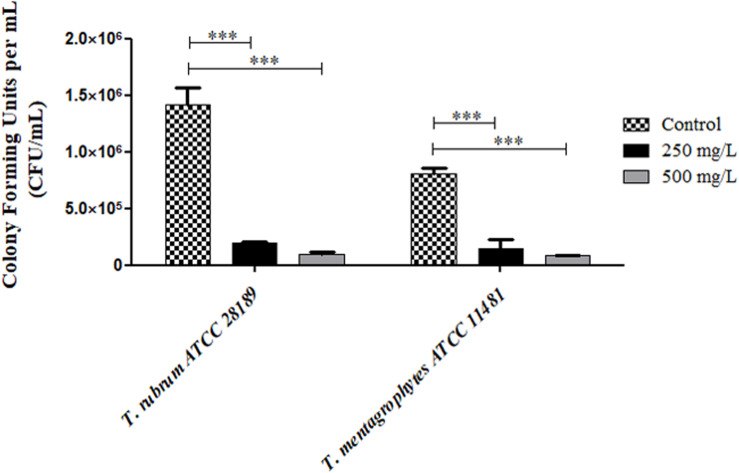
Values of colony-forming units per mL (CFU/mL) recovered from mature biofilms formed by the strains of *T. rubrum* ATCC 28189 and *Trichophyton mentagrophytes* ATCC 11481 treated with nonyl incorporated into the nanostructured lipid system (NLS). The treatment significantly reduced the colony-forming units when compared to the control without treatment (***p < 0.0001).

To confirm the findings of SEM, a biofilm colony count test was performed after treatment with the nanostructured compound in concentrations of 250 and 500 mg/L. In this assay, strains of *T. rubrum* ATCC 28189 and *T. mentagrophytes* ATCC 11481 were used. The treatment significantly reduced the colony-forming units in both strains and concentrations tested (*p* < 0.0001), proving the anti-biofilm action of nanostructured compound (Figure 7).

## Discussion

In recent years, microorganisms have developed considerable resistance to commercially available drugs. Within this context, many studies have shown that nanoparticles (NPs) can overcome the mechanisms of resistance to antimicrobials, such as decreased absorption, overexpression of efflux pumps, biofilm formation, among others (Voltan et al., 2016). In addition, the treatment of many diseases, including some mycoses, presents a common problem, which is the difficulty of some drugs in reaching the target of action, as they may have restricted efficacy, lack of selectivity, and low solubility. In view of this problem, the development of nanomaterials can overcome these limitations and inconveniences and offer the development of drugs in a sustained, controlled and oriented way to avoid associated adverse systemic effects (Landriscina et al., 2015).

The present work shows that the incorporation of a promising compound with broad spectrum antifungal activity into a nanostructured lipid system allowed the maintenance of its effectiveness for both planktonic cells and dermatophyte biofilms and reduced their toxicity in human epithelial monolayers and alternative animals such as *C. elegans* and zebrafish. The NLS characterization tests showed that the incorporation of nonyl into the NLS caused a small variation in the size of the particles. The values obtained are in the range from 10 to 250 nm (100–2500 Å), which is considered an ideal range for nanostructured lipid systems of the micro-emulsion type, according to Formariz et al. (2005) and Singulani et al. (2018). When comparing the control formulation (NLS) and the formulation containing the incorporated compound (NLS + nonyl), there is a small increase in the size of the particles, which is a strong indication that the sample has been incorporated into the lipid system. With regard to the PDI, index that shows the relative homogeneity between the particle sizes distributed in the measured sample, the values obtained (0.331 ± 0.020 and 0.377 ± 0.004) are in the range considered adequate (up to 0.5), and allow to infer that the samples have a certain homogeneity. The lower the PDI, the greater the uniformity of the particles (Goyal et al., 2012). For the zeta potential, the negative charge obtained comes from the components of the formulations, such as cholesterol, which in its structure has a free hydroxyl group [OH −]^–^ and soy phosphatidylcholine, which has free ester groups [−OC = O −]^–^ (Silva et al., 2016).

The susceptibility tests show a variation in MIC values between the free and nonyl incorporated to the NLS of only one dilution for *T. rubrum* and eight dilutions for *T. mentagrophytes*. Some authors claim that there is no significance in varying up to two dilutions in microdilution assays (Pfaller et al., 2011; Singulani et al., 2018). Regarding the nonyl compound 3,4-dihydroxybenzoate, the influence of the hydrophobic carbon chain on antifungal activity has been demonstrated, in addition to the fact that its antimicrobial effect increases with the addition of the side alkyl chain (Soares et al., 2014). However, for dermatophytes, from more than 10 carbons, there is a reduction or inhibition of this antifungal activity, resulting from the increase in the size of the molecule, which makes it difficult to cross through the fungal cell wall and the plasma membrane.

A study conducted by Medina-Alarcón et al. (2017), proved the broad spectrum of protocatechuic acid derivatives and their excellent activities against fungi of the genus *Paracoccidioides*. The derivative with 12 carbons (dodecyl – DOD) in the side alkyl chain was incorporated into a lipid nanostructured system using another type of non-ionic surfactant containing castor oil and polyoxyl-60/polyethylene glycol-hydrogenated. The results showed that the incorporation maintained the antifungal activity, with considerable variation; however, NLS + DOD increased the inhibition of the fungus adhesion to cell monolayers and components of the extracellular matrix.

Several studies have demonstrated the effectiveness and even the improvement of MIC values after the incorporation of compounds or drugs in lipid systems and micro-emulsions. Bonifácio et al. (2015) incorporated extracts from stems and leaves of *Astronium fraxinifolium*, *Astronium graveolens*, and *Astronium urundeuva* into NLS and tested its activity against *Candida albicans*. The extracts alone did not show antifungal activity, but after being incorporated they were able to inhibit fungal growth, with MIC values ranging from 15.6 to 250 μg/mL. The authors believe that the improved solubility of the extracts after incorporation is directly related to the inhibition of the growth of the microorganism. Guimarães et al. (2014) incorporated a thiophene-derived compound (5CN05) in microemulsions and tested its effectiveness against yeasts of the genus *Candida* and *C. neoformans*. There was a decrease in MIC values, with more significant values for *C. neoformans*. The authors believe that these results may be a consequence of the composition of the microemulsion, since the presence of surfactants and the oil phase may promote a better diffusion of the compound or drug, enhancing its permeation. The same was stated by El-Hadidy et al. (2012) and Sato et al. (2017) by incorporating the drug voriconazole and the antifungal complexes of the microemulsions and copper nanostructured lipid carriers, respectively, showed that the antimicrobial activity increased significantly.

The assays on keratinocyte monolayers proved the ability to reduce toxicity when nonyl was incorporated into the NLS. Similar results were obtained by Medina-Alarcón et al. (2017) with dodecyl protocatechuate in pneumocyte cell lines (MRC5) and human hepatoma cells (HepG2). In a similar study, Dananjaya et al. (2018) investigated the antifungal activity and cytotoxicity of ZnO-chitosan (ZnO-C) nanocomposites against *Candida albicans* and in type 2 human epithelium cells (HEp-2), and observed that ZnO-C nanoparticles were able to decrease toxicity in these monolayers. Singh et al. (2017) verified the toxicity of copper oxide nanoparticles and free copper oxide in human fibroblasts (HDF) and in human hepatoma cells (HepG2). The results showed that the nanoparticles were no toxic to HDF cells, however, the toxicity was increased in the HepG2 monolayers. Regarding drugs, the greatest and best example of reducing toxicity through incorporation into nanostructures is amphotericin B (AmB). AmB is a reference for the treatment of invasive fungal infections. Its administration results in several adverse effects, the most severe being nephrotoxicity. In order to reduce its intrinsic toxicity and, consequently, these adverse effects, its liposomal form was developed. The literature reports that patients treated with non-liposomal AMB, in more than 65% of cases, had adverse effects after administration, with an incidence of nephrotoxicity between 12 and 50%. In contrast, the administration of liposomal amphotericin B halved the incidence of side effects (20% to 40% of cases), as well as nephrotoxicity (between 9 and 25%) (Steimbach et al., 2017). Other studies conducted by Johansen and Gotzsche (2014), demonstrate that liposomal amphotericin B was also able to decrease nephrotoxicity in a restricted population of neutropenic cancer patients.

Regarding *in vivo* tests, the use of mammals for this purpose has become restricted for ethical reasons, high costs, new animal protection requirements, always considering the three R’s (Refinement, Reduction and Replacement). *C. elegans* is a geophilic, hermaphrodite or male nematode, which measures about 1 mm, feeds on bacteria and has been an alternative to study the efficacy and toxicity of new compounds with antimicrobial properties, in addition to host-pathogen interactions (Scorzoni et al., 2013; Medina-Alarcón et al., 2017). Our results showed a difference of strains AU37 and N2 in response to treatment with nonyl incorporated to NLS, in which the mutant strain AU37, proved to be more sensitive. This strain is a double mutant and has been widely used in the interaction between *C. elegans* and fungi, because its glp-4 mutation makes the larvae unable to reproduce at 25°C and the sek-1 mutation increases the sensitivity to various pathogens (Scorzoni et al., 2013; Singulani et al., 2017). This sek-1 allele encodes a mitogen-activated protein kinase (MAP) and is involved in the pathogen resistance pathway, which is necessary for the innate immunity of *C. elegans* larvae. This mutation, in addition to making the larvae more sensitive to pathogens, also results in a slower locomotion phenotype of the larvae. Some studies report that this mutation together with some substances increases the longevity of the larvae (Kim et al., 2002; Saul et al., 2010; Yu et al., 2014; Hoyt et al., 2017).

Zebrafish (*D. rerio*) is a species of conventionally ornamental fish, belonging to the Cyprinidae family and distributed in several countries around the world. In recent years there has been an increase in studies showing that zebrafish is a potential biological model for studies of toxicity and teratogenic effects also in human diseases (Kalueff et al., 2014; Chakraborty et al., 2016; Genge et al., 2016; Morales and Wingert, 2017). This model presents exceptional characteristics for an alternative for *in vivo* studies, such as easy maintenance, rapid development, high rate of oviposition and fertility, not to mention the transparency of the embryo that allows real-time visualization of changes over time (Chakraborty et al., 2016). Furthermore, it has a toxicological and teratogenic profile predictive of the human profile (Singulani et al., 2017). Recent studies have shown that zebrafish is ideal for assessing the toxicity of nanoparticles (Chakraborty et al., 2016). Our results showed that the incorporation of nonyl into NLS reduced toxicity in the embryos by 12.7 times, showing an increase in the therapeutic margin through incorporation. Similar results were obtained by Singulani et al. (2018), when the compound derived from gallic acid, dodecyl gallate was incorporated into an NLS using another surfactant, Brij^®^ 35.

In the susceptibility tests of mature biofilms against the nanostructured compound, the use of additional tests was necessary, since the incongruity of the XTT reduction assay have been proven. There are some reports in the literature about the limitations of using XTT (Chandra et al., 2008; Costa-Orlandi et al., 2017). However, one in particular report conflicts of cell viability in studies using MTT and XTT and nanoparticles. The authors believe that both MTT and XTT, when in contact with some nanoparticles, underestimate toxicity by overestimating cell viability (Wang et al., 2011). The activity of nanostructures on microbial biofilms has been reported in the literature (Tian et al., 2017; Mi et al., 2018). Thaya et al. (2016) demonstrated the ability of silver and zinc nanocomposites (CS/Ag/ZnO) based on chitosan to damage mature biofilms, as well as inhibit their maturation. The same was reported by Rajkumari et al. (2017) with the hordenine phytocomposite incorporated into gold nanoparticles.

Unlike that observed in the XTT assay, the images obtained by SEM revealed an extensive tangled of collapsed hyphae showing the potent antibiofilm action of the nanostructured nonyl, suggesting that the XTT assay results test may have been overestimated as reported by Wang et al. (2011). Finally, the results of counting colony-forming units showed the potent anti-biofilm effect of nanostructured nonyl, with the eradication of about 83–86% cells when the biofilms were treated at a concentration of 250 mg/L and 90–94% cells when treated with a concentration of 500 mg/L, confirming the potent action of nonyl incorporated into the NLS against mature dermatophyte biofilms. With these results we can affirm that nonyl incorporated into the NLS containing the surfactant Brij98 is extremely promising for the treatment of dermatophytosis and specially for infections with involvement of biofilms.

## Conclusion

The results generated in this work emphasize the importance of considering biofilms in the discovery of new compounds with antimicrobial properties, since high concentrations are necessary for the extermination of these extremely intelligent and dynamic communities of microorganisms. In addition, the incorporation of the compound nonyl 3,4-dihydroxybenzoate to NLS has proved to be an effective alternative for maintaining its effectiveness, increasing its solubility and reducing its toxicity both *in vitro* and *in vivo*. Additional studies with nonyl, including its likely mechanisms of action, are in the process of being submitted for publication, once it is a very promising compound. In any case, this work opens doors for the continuation of studies for this compound or others can evolve in the antifungal pipeline, becoming serious candidates for drugs in the future.

## Data Availability Statement

The datasets generated for this study are included in the article.

## Ethics Statement

The housing and breeding of the fish used in this study received appropriate national and institutional approvals (National Council for Animal Experimentation Control – CONCEA and Faculty of Pharmaceutical Sciences, permit number 01.0082.2014).

## Author Contributions

CC-O, NB, and AS-P drafted the manuscript. CC-O, NB, AS-P, JB, CS, PS, and JS performed the experiments. LS supervised the experiments with *C. elegans*. MC, LR, and AN synthesized and provided the nanostructured lipid systems and nonyl, respectively. CC-O, AF-A, and MM-G designed and supervised the project. All authors participated in data analysis and critical revision of the manuscript and approved the final version.

## Conflict of Interest

The authors declare that the research was conducted in the absence of any commercial or financial relationships that could be construed as a potential conflict of interest.
